# Chylorrhea following laparoscopy assisted distal gastrectomy with D1+ dissection for early gastric cancer: A case report^☆^

**DOI:** 10.1016/j.ijscr.2013.10.006

**Published:** 2013-10-29

**Authors:** Takanobu Yamada, Yasuyuki Jin, Kimiatsu Hasuo, Yukio Maezawa, Yuta Kumazu, Yasushi Rino, Munetaka Masuda

**Affiliations:** a Department of Surgery, Hadano Red Cross Hospital, 1-1 Tatenodai, Hadano, Kanagawa 257-0017, Japan; b Department of Surgery, Yokohama City University, 3-9 Fukuura, Kanazawa, Yokohama, Kanagawa 236-0004, Japan

**Keywords:** Chylorrhea, Laparoscopic gastrectomy, LADG, Postoperative complication

## Abstract

**INTRODUCTION:**

Chylorrhea is a form of lymphorrhea involving digested lipid products absorbed in the small intestine. Here we report a rare case of chylorrhea after laparoscopy-assisted distal gastrectomy (LADG) with D1+ dissection that resolved following administration of a low-fat diet.

**PRESENTATION OF CASE:**

A 35-year-old woman with early gastric cancer underwent LADG with D1+ dissection, and on postoperative day 4, the drain output increased and the fluid with a high triglyceride level (740 mg/dL) changed from clear to milky. On postoperative day 6, oral intake of a low-fat diet was initiated after a 2-day fast, and the daily drain output decreased from postoperative day 9. The drain tube was withdrawn on postoperative day 15, and the patient was discharged on postoperative day 17.

**DISCUSSION:**

D1+ dissection does not typically cause injury to the lymphatic trunks, cisterna chyli, or thoracic duct. The maximum output of chylous ascites was minimal, and thus, we assumed that chylorrhea occurred from slightly injured lymphatics with anatomical variation.

**CONCLUSION:**

Chylorrhea after LADG with D1+ dissection is very rare. The fasting of our case followed by a low-fat diet without TPN would be an effective therapy. As a result, our case recovered favorably without further therapy.

## 1 Introduction

Lymphorrhea is the leakage of lymphatic fluid in response to lymphatic duct disturbances due to malignant neoplasms, blunt abdominal trauma, bacterial peritonitis, pelvic irradiation, peritoneal dialysis, and abdominal tuberculosis. It is sometimes reported after aortic surgery, spinal surgery, and lymph node dissection for various malignancies.^1^ Chylorrhea is a form of lymphorrhea involving digested lipid products absorbed in the small intestine. The incidence of chylorrhea after gastrectomy with D2 or less dissection is reportedly only 0–0.4%, and there have been few cases associated with D1 dissection.^2,3^ There are some reports regarding lymphorrhea after gastrectomy and a few case reports on chylorrhea^4–6^ with extended lymph node dissection; however, until now, there are no reports describing chylorrhea after laparoscopy-assisted distal gastrectomy (LADG) with D1+ dissection.^3^ Here we describe a rare case of chylorrhea after LADG with D1+ dissection that was resolved by administration of a low-fat diet.^3^

## 2 Presentation of case

**Fig. 1:**
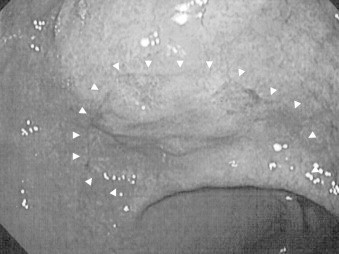
Appearance of upper gastrointestinal endoscopy. White arrow heads show 0-IIc lesion at lesser curve of antrum.

A 35-year-old woman was admitted to our hospital for treatment of gastric cancer in March 2012. Preoperative endoscopy revealed a 2.0 cm × 1.5 cm depressed-type (0-IIc) lesion in the lower third of the stomach that was histologically diagnosed as signet ring cell carcinoma (Fig. 1). Preoperative examination revealed no regional or distant lymph node metastasis. The patient underwent LADG with D1+ dissection using ultrasonic coagulating shears.^3^ Billroth I anastomosis surgery was selected for reconstruction, and a drain was placed under the left liver lobe from the right upper abdomen. The pathological staging was T1a (M), N0 (0/51), H0, P0, M0, and stage IA according to the *Japanese Classification of Gastric Carcinoma* (3rd English edition).^7^ Pathological analysis showed a predominance of poorly differentiated adenocarcinoma.

**Fig. 2:**
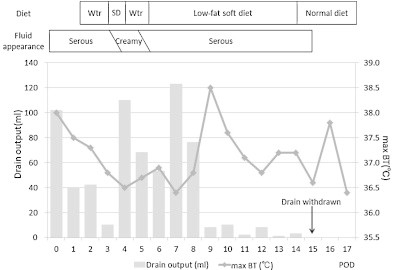
Clinical course. Wtr, water; SD, soft diet; BT, body temperature.

**Fig. 3:**
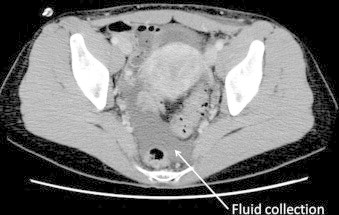
A contrast-enhanced CT scan shows a little ascites at pelvic space (white arrow).

On postoperative day 4, the patient was allowed oral intake of food (Fig. 2), but 3 h after she ate food, the drain output increased with the fluid, which had a high triglyceride level (740 mg/dL), changing from clear to milky. Computed tomography revealed collected fluid in the pelvic space and a small amount in the upper abdominal space (Fig. 3), and thus, we immediately discontinued her oral intake; the drain discharge became clear again. On postoperative day 6, oral intake with a low-fat diet (1 g/day) was initiated. The daily drain output decreased from postoperative day 9, and we gradually increased the fat content of the diet by 1–35 g/day from postoperative day 12. The drain tube was removed on postoperative day 15, and the patient was discharged on postoperative day 17. Ten months after surgery, she had no signs of chylorrhea and her postoperative general condition was good.

## 3 Discussion

A consensus on the definition of chylorrhea has not been reached; however, Griniatsos et al. proposed the criteria for diagnosis of chylorrhea to be the presence of a non-bloody, amylase- and bilirubin-free, triglyceride-rich, milky or creamy, peritoneal fluid in the drainage tube or on aspiration from postoperative day 3 onward, independent of the amount of fluid drained daily.^4^ In our case, a milky fluid was present immediately after restart of oral intake on postoperative day 4, and this fluid contained a relatively high triglyceride level that resolved when the diet was withdrawn.

Digestion of long-chain fatty acids and lipid soluble vitamins absorbed from lymphatic capillaries occurs in the small intestine. The flow of lymphatic fluid follows a route from the prenodal collecting lymphatics to the lymph nodes, postnodal collecting lymphatics, lymphatic trunks, cisterna chyli, and finally to the thoracic duct, which is a one-way transportation system because of the smooth muscle and valves of the collecting lymphatics. Griniatsos et al. described the possible causes of chylorrhea after gastrectomy with basic dissection as follows: (i) disruption at the level of confluence in the abdominal lymph trunks, as opposed to the cisterna chili; (ii) concentrated lymphatic fluid in the collecting lymphatics in some instances; and (iii) anatomical variations at the origin of the confluence of the abdominal lymph trunks or the cisterna chyli itself.^4^ We guess that the cause of chylorrhea in this case was anatomical variation, because D1+ dissection does not typically cause injury to the lymphatic trunks, cisterna chyli, or thoracic duct.^3^ The maximum output of chylous ascites was only 123 mL/day, and thus, we assumed that the chylorrhea had occurred from slightly injured abnormal lymphatic.

The essential therapy for lymphorrhea, including chylorrhea, is total parental nutrition (TPN) without fat infusion and fasting.^6,8^ TPN can decrease lymph flow in the thoracic duct from 220 mL/kg/h to 1 mL/kg/h and can compensate for nutritional deficits.^8^ A diet containing medium chain triglycerides (MCTs) (6–12 carbons) can reduce chyle flow and maintain proper nutrition because MCTs are directly transported into intestinal cells. Therefore, Cardenas et al. recommended using a low-fat diet with MCTs as a first line therapeutic option^9^; however, this chylorrhea therapy remains controversial.^4^ Some recent reports indicated that somatostatin or octreotide administration was an effective treatment against chylorrhea, but the detailed mechanisms have not been clarified.^10^ If nonsurgical therapy is not effective, diagnostic imaging such as lymphangiography and surgical exploration are recommended.^4,8^ A review by Aalami et al. found that 67% of chylorrhea cases were treated successfully with conservative procedures and 33% were treated successfully via surgery.^8^

## 4 Conclusion

Chylorrhea after LADG with D1+ dissection is very rare, because that procedure does not typically cause injury to the center of lymphatic system. The fasting of our case followed by a low-fat diet without TPN would be an effective therapy. As a result, our case recovered favorably without further therapy.

## Conflict of interest

Takanobu Yamada and other coauthors have no conflict of interest.

## Funding

None.

## Ethical approval

Written informed consent was obtained from the patient for publication of this case report and accompanying images. A copy of the written consent is available for review by the Editor-in-Chief of this journal on request.

## Author's contribution

Takanobu Yamada, Yasuyuki Jin, Kimiatsu Hasuo, Yukio Maezawa, and Yuta Kumazu are contributed in clinical treatment of the patients.

Takanobu Yamada, Yasushi Rino, and Munetaka Masuda are contributed in writing, editing, and revising manuscript.
